# Computationally derived compound profiling matrices

**DOI:** 10.4155/fsoa-2018-0050

**Published:** 2018-07-24

**Authors:** Martin Vogt, Swarit Jasial, Jürgen Bajorath

**Affiliations:** 1 Department of Life Science Informatics, B-IT, LIMES Program Unit Chemical Biology and Medicinal Chemistry, Rheinische Friedrich-Wilhelms-Universität, Endenicher Allee 19c, D-53113 Bonn, Germany

**Keywords:** biological screening, compound profiling matrices, computational design, open access data, targets, test compounds

## Abstract

**Aim:**

Screening of compounds against panels of targets yields profiling matrices. Such matrices are excellent test cases for the analysis and prediction of ligand–target interactions. We made three matrices freely available that were extracted from public screening data.

**Methodology:**

A new algorithm was used to derive complete profiling matrices from assay data.

**Data:**

Two profiling matrices were derived from confirmatory assays containing 53 different targets and 109,925 and 143,310 distinct compounds, respectively. A third matrix was extracted from primary screening assays covering 171 different targets and 224,251 compounds.

**Next steps:**

Profiling matrices can be used to test computational chemogenomics methods for their ability to predict ligand–target pairs. Additional matrices will be generated for individual target families.

## Lay abstract

Screening of a given number of small molecules in different assays produces a so-called profiling matrix. This matrix reports for each compound inactivity or activity in all assays. Such profiling matrices are frequently produced in the pharmaceutical industry but rarely disclosed. We have recently reported a computational methodology to derive such matrices from independently conducted biological assays. Herein, we describe three large profiling matrices we have extracted from many experimental screens and made publicly available. These matrices should be helpful to investigators studying the interactions of small molecules with different biological targets.

## Graphical abstract

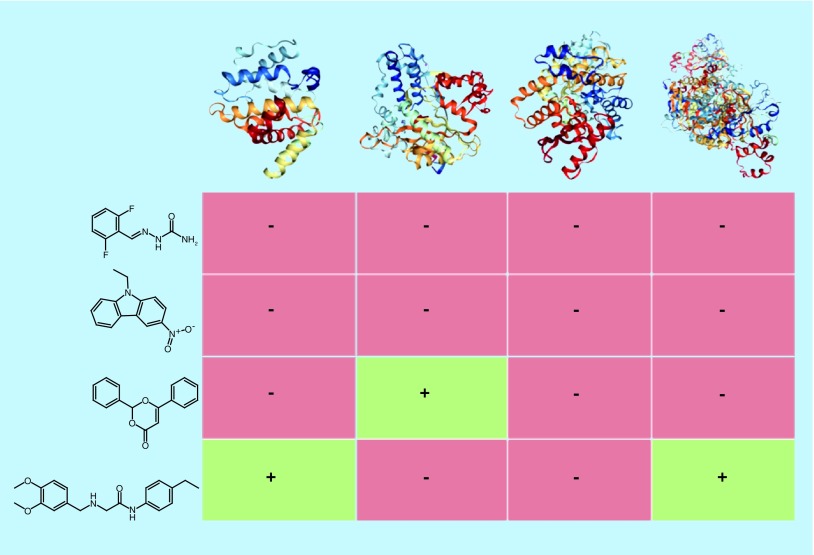

Shown is a small compound profiling matrix resulting from assaying four compounds (rows) against four target proteins (columns). ‘+’ and ‘−’ signs denote compound activity and inactivity, respectively.

In compound profiling, collections of small molecules are assayed against arrays of targets [1,2]. In the resulting data structure, termed profiling matrix, rows correspond to compounds and columns to targets, respectively, as illustrated in Figure 1A. Targets for compound profiling might be closely related (e.g., members of a given protein family), distantly related or unrelated. Furthermore, one particular assay format might be used, especially for closely related targets, or different assays might be combined to generate a matrix. Experimental matrices are often incomplete or sparse, which means that not all compounds have been tested against all targets. Profiling campaigns are mostly – but not exclusively – carried out in the pharmaceutical industry, but the results are rarely disclosed. Only a few compound profiling experiments have been published during the past decade and almost all of them targeted protein kinases [1–6].

**Figure 1.**
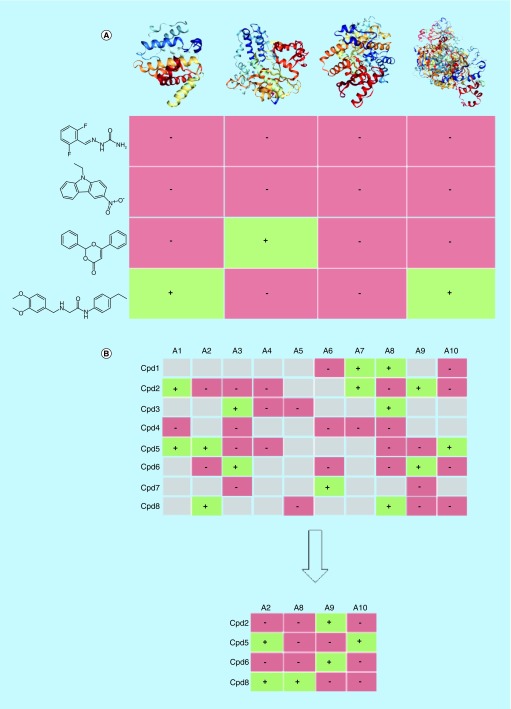
**Profiling matrices.** **(A)** A small prototypic compound profiling matrix (taken from a larger matrix) is shown consisting of four compounds (rows) tested against four targets (columns). These targets include, from the left to the right, Rac GTPase-activating protein 1, DNA polymerase beta, pyruvate kinase and lysine-specific demethylase 4A. Green matrix cells with a ‘+’ report activity of a compound against a target and red matrix cells (‘-’) report inactivity. **(B)** The extraction of a complete profiling matrix (bottom) from a set of assays (top) is schematically illustrated. The matrix consists of eight compounds (Cpd1–Cpd8) and ten targets (A1–A10). In the sparse assay matrix (top), gray cells indicate that a compound was not tested against a target.

In addition to exploring compound activity or selectivity, profiling matrices are also of high interest for the systematic analysis of ligand–target interactions, which represents the core of chemogenomics [7,8]. Ultimately, chemogenomics efforts aim at generating a global ligand–target matrix accounting for as many interactions as possible. Since it will hardly be feasible to assess all possible ligand–target interactions experimentally, computational prediction of interactions also plays an important role in chemogenomics [8,9]. For this purpose, learning from profiling matrices becomes particularly attractive, but it is hampered by data incompleteness or sparseness [10]. The utility of sparse matrices to derive predictive computational models is limited. By contrast, dense or complete profiling matrices provide a much improved basis for training and evaluating machine learning models to predict ligand–target interactions. Furthermore, using such matrices, the outcome of screening experiments can be predicted under realistic conditions, for instance, in the presence of many consistently inactive and small numbers of active compounds. This distribution leads to highly unbalanced data sets, which is a difficult scenario for machine learning.

Given the very limited availability of compound profiling matrices in the public domain, we have developed a computational methodology for extracting complete or dense profiling matrices from high-throughput screening data for diverse targets [11]. The algorithm was applied to generate large matrices for evaluating the ability of machine learning approaches to predict the outcome of screening experiments [12].

Herein, three profiling matrices are described and made freely available for computational chemogenomics and other applications.

## Methodology

The new computational method developed for the extraction of profiling matrices from large volumes of screening data [11] is a variant of biclustering algorithms [13]. In their simplest form, these algorithms attempt to generate biclusters (submatrices) with constant or nearly constant values from an original matrix, for instance, by iterative removal of rows and/or columns from the matrix. Biclustering is frequently used for the analysis of expression data to identify genes with similar expression levels [13].

A set of biological assays using overlapping compound collections can be organized as a sparse assay-compound matrix in which rows and columns represent screening compounds and assays, respectively. Cells in this matrix account for three different test categories. They either report detected activity (in other words, a compound was active in a given assay), inactivity or the absence of experimental data (a compound was not tested in any assay). The latter category determines the sparseness of the matrix.

The algorithm (full details are provided in [11]) iteratively removes compounds or assays from a sparse matrix to generate submatrices with gradually increasing density of cells containing test data. During each iteration, the column or row with the lowest density is removed. Thereby, from a sparse matrix, a complete profiling matrix is ultimately obtained where each compound is tested in each assay, as illustrated in Figure 1B. The complete matrix captures experimentally observed hit rates across all assays.

The initial goal of iterative removal of compounds or assays is retaining a maximally sized complete submatrix (100% density). In addition, weights can be applied to preferentially retain assays (at the cost of removing compounds) or compounds (at the cost of removing assays). This weighting scheme leads to a relative enrichment of assays over compounds or vice versa while reducing matrix size and transforming the complete submatrix into dense matrices (e.g., with 95% density). Such dense submatrices balance compound and assay coverage in a desired and controlled manner. Another weighting factor can be applied to preferentially retain active matrix entries or assays with above-average hit rates, which leads to a relative enrichment of active compounds in dense submatrices.

## Data

Profiling matrices were extracted from PubChem BioAssays [14]. Two matrices were derived from confirmatory (dose–response) screening assays. Importantly, these matrices were designed to contain the same targets, but nonoverlapping sets of screening compounds (in other words, they did not share any compound). In addition, a complete profiling matrix of very large size was generated from primary (single-dose) screening assays.

### Source assays

From PubChem, confirmatory and primary screening assays were assembled. Confirmatory assays typically yield AC_50_ values from dose-response measurements. Screening compounds were only selected if they were explicitly designated as ‘active’ or ‘inactive’ and compounds with detectable assay interference potential [15] were removed. For targets with multiple assays, the one containing the largest number of tested compounds was retained. On the basis of these criteria, 625 confirmatory assays containing 422,105 compounds were selected. Each assay represented a unique target (individual protein).

The initial density of the resulting sparse assay matrix was only 11%. Cells with experimental data included 1.15% activity annotations. Thus, the fraction of active entries in the sparse matrix was 0.11×1.15% = 0.13%. From these assay data, two profiling matrices were algorithmically extracted.

A corresponding protocol was applied to select primary screening assays, which test a single compound concentration and report the percentage of inhibition or residual activity. The selection yielded 476 primary assays and 767,895 compounds. The density of the resulting sparse assay matrix was 24%. In this case, cells with active entries included 0.67% activity annotations, corresponding to a fraction of 0.24 × 0.67% = 0.16% of all entries.

For all matrices, binary activity annotations were generated from curated screening data since activity measurements were assay-dependent, and hence not transferable across different assays. This is a general requirement for profiling matrices combining different assay formats.

### Computationally derived profiling matrices

#### Matrix 1

First, a complete matrix (density 100%) was extracted from the selected confirmatory assays comprising 53 assays (targets) and 110,636 compounds. Molecular structures were retrieved from PubChem and an in-house curation protocol was applied to remove duplicates, yielding a final set of 109,925 unique compounds. In matrix 1, all cells contained binary annotations of activity or inactivity. The 53 assays included 46 assays with a hit rate of less than 1% and four that did not produce any hits.

#### Matrix 2

Second, a larger dense matrix was generated from the same 53 assays with a density adjustment to favor compound coverage. From this matrix, all compounds contained in matrix 1 were removed. The density of the resulting matrix 2 was 96% and it contained 143,310 unique compounds after removal of duplicates. The 53 targets contained in matrix 1 and 2 are provided in our data deposition specified below.

#### Matrix 3

From the selected primary assays, a large complete matrix with 100% density was extracted comprising 171 assays (targets) and 224,251 unique compounds (after removing duplicates). So far, matrix 3 has not been subjected to machine learning studies (and was not investigated in [12]).

### Distribution of active & inactive compounds

Matrix 1 contained 105,475 (96.0%) and matrix 2 contained 110,218 (76.9%) compounds that were consistently inactive in all assays. Matrix 1 included 3639 (3.3%) compounds with single-target and 811 (0.7%) compounds with multi-target activity. For matrix 2, the corresponding numbers (percentages) were 19,069 (13.3%) and 14,023 (9.8%) compounds. Matrix 1 and matrix 2 contained 0.1% and 0.8% of cells with activity annotations, respectively. Thus, the composition of these matrices was by design highly unbalanced, capturing experimental readouts of confirmatory screening. Thus, they represent realistic data structures for machine learning and activity predictions.

Matrix 3 contained 119,192 (53.2%) compounds that were consistently inactive in all assays, 57,215 (25.5%) compounds with single-target and 47,844 (21.3%) compounds with multi-target activity. Furthermore, 0.6% of the cells had activity annotations.

### Data format & open access deposition

For public release, matrices 1, 2 and 3 were stored in comma separated values (CSV) format, which is widely used to store tabular data and provides easy import into a variety of programs. PubChem compound identifiers were reported in rows and assay identifiers in columns. Activity information was assigned to matrix cells using values of 1 or 0, representing activity and inactivity, respectively. In matrix 2, cells not containing experimental data were designated ‘NA’. Separate tables were provided, listing the targets contained in matrix 1, 2 and 3, respectively. Formatted matrix 1, 2, and 3 have been made freely available as a deposition on the ZENODO open access platform [16].

## Limitations & next steps

Currently, the only limitation in generating complete or dense profiling matrices is access to sufficiently large volumes of screening data. Of course, the quality of the original data – or lack thereof – inevitably determines, and potentially limits, the utility of computed matrices. Hence, care must be taken to curate available data to the extent possible.

We have applied matrices 1 and 2 for activity prediction using a variety of machine learning methods including deep learning [12]. Given their inherent experimental unbalance, these matrices were found to be challenging test cases, yielding varying prediction accuracy, depending on the assays.

Going beyond assay-based activity predictions, these matrices can also be used to predict ligand–target pairings across assays, a primary chemogenomics application.

Matrix 3 was extracted from primary screening data to obtain a complete matrix of very large size. Compared with matrices 1 and 2, matrix 3 covers more than three-times as many targets and contains about as many compounds as matrices 1 and 2 combined. Because matrix 3 was generated from primary assays the confidence level of its activity annotations is lower than for matrix 1 and matrix 2. This needs to be taken into consideration when applying matrix 3 in a similar fashion to matrices 1 and 2. It will be interesting to see how predictive chemogenomics models will perform under experimental conditions captured by matrices 1, 2 and 3, respectively; the jury is still out.

The next step in matrix design will be the derivation of profiling matrices for prominent target families such as G-protein-coupled receptors, proteases or kinases. Members of target families often have overlapping yet distinct compound activity profiles. Distinguishing between such profiles provides another interesting test scenario for computational chemogenomics. Again, data availability will be the only limiting factor in deriving target family-based profiling matrices. New matrices will also be made available in future updates of our open access deposition.

**Executive summary**

- The lack of publicly available compound profiling data is emphasized.
- The utility of profiling matrices for machine learning and computational chemogenomics is highlighted.

**Methodology**

- A new methodology for the computational extraction of complete or dense profiling matrices from screening data is described.
- Different matrix design strategies are discussed.

**Data**

- Two exemplary and nonoverlapping profiling matrices from confirmatory screening data covering the same targets are detailed.
- In addition, a large complete matrix from primary screening data is introduced.
- An open access deposition containing these matrices in a convenient and easily accessible format is described.

**Limitations & next steps**

- Data availability- and quality-based limitations of matrix generation are discussed.
- Different strategies for computational modeling of matrices are outlined.
- The derivation of target family-based matrices is considered as a next step in matrix design.
